# An Italian Neurology Outpatient Clinic Facing SARS-CoV-2 Pandemic: Data From 2,167 Patients

**DOI:** 10.3389/fneur.2020.00564

**Published:** 2020-05-29

**Authors:** Carla Piano, Enrico Di Stasio, Guido Primiano, Delfina Janiri, Marco Luigetti, Giovanni Frisullo, Catello Vollono, Matteo Lucchini, Valerio Brunetti, Mauro Monforte, Valeria Guglielmi, Giacomo Della Marca, Amelia Evoli, Camillo Marra, Massimiliano Mirabella, Davide Quaranta, Enzo Ricci, Serenella Servidei, Gabriella Silvestri, Simone Bellavia, Sara Bortolani, Francesco Bove, Riccardo Di Iorio, Andrea Di Paolantonio, Danilo Genovese, Tamara Ialongo, Maria Rita Lo Monaco, Jessica Marotta, Agata Katia Patanella, Alessia Perna, Martina Petracca, Giorgia Presicce, Vittorio Riso, Eleonora Rollo, Angela Romano, Marina Romozzi, Cristina Sancricca, Irene Scala, Gregorio Spagni, Marcella Solito, Luca Tricoli, Paola Zinzi, Paolo Calabresi, Anna Rita Bentivoglio

**Affiliations:** ^1^Dipartimento Scienze dell'Invecchiamento, Neurologiche, Ortopediche e della Testa-Collo, Fondazione Policlinico Universitario A. Gemelli IRCCS, Rome, Italy; ^2^Dipartimento di Scienze Biotecnologiche di Base, Cliniche Intensivologiche e Perioperatorie, Università Cattolica del Sacro Cuore, Rome, Italy; ^3^Department of Psychiatry and Neurology, Sapienza University of Rome, Rome, Italy; ^4^Dipartimento di Neuroscienze, Università Cattolica del Sacro Cuore, Rome, Italy; ^5^Centro Clinico, NEMO, Rome, Italy

**Keywords:** neurology, infection, coronavirus, pandemic, health care, COVID-19, personalized medicine

## Abstract

**Objective:** Neurological sequelae of SARS-CoV-2 infection have already been reported, but there is insufficient data about the impact of the pandemic on the management of the patients with chronic neurological diseases. We aim to analyze the effect of COVID-19 pandemic and social restriction rules on these fragile patients.

**Methods:** Patients with chronic neurologic diseases routinely followed at the outpatient clinic of Gemelli University Hospital, Rome, were assessed for symptoms suggestive of SARS-CoV-2 infection in the pandemic period, consequences of social restrictions, and neurological disease features, concomitant medical conditions, current medical and disease-specific treatments. Data source: a dedicated telephone survey designed to encompass questions on COVID-19 symptoms and on pandemic effects in chronic neurologic conditions.

**Results:** Overall, 2,167 individuals were analyzed: 63 patients reported contact with COVID-19 positive cases, 41 performed the swab, and 2 symptomatic patients tested positive for COVID-19 (0.09%). One hundred fifty-eight individuals (7%) needed urgent neurological care, deferred due to the pandemic; 641 patients (30%) suspended hospital treatments, physiotherapy or other support interventions; 405 individuals (19%) reported a subjective worsening of neurological symptoms.

**Conclusions:** In our population, the presence of neurological chronic diseases did not increase the prevalence of COVID-19 infection. Nevertheless, the burden of neurological disorders has been worsened by the lockdown.

## Introduction

The coronavirus disease 2019 (COVID-19) has spread across Italy since the end of February 2020 and resulted in an increase in total deaths of nearly 100% (1). On March 11th, the World Health Organization (WHO) characterized the novel severe acute respiratory syndrome coronavirus-2 (SARS-CoV-2) outbreak as a pandemic (2). In response to this, the Italian Government implemented a series of emergency containment measures, including the restriction of social contacts and the quarantine of COVID-19 positive and suspect cases.

The principal manifestations of COVID-19 are fever, cough and dyspnea; the most severe complication of the infection is the acute respiratory distress syndrome (3). In a recent editorial Manji et al. (4) highlighted the concern of neurologists for vulnerability to COVID-19 in patients with neurological diseases. Patients on disease-modifying and immunosuppressant treatments and with respiratory impairment from neuromuscular weakness might be particularly at risk for severe COVID-19 complications (5). Nevertheless, there is insufficient data available on the outcomes of patients with pre-existing neurological disorders (4) or on the impact of the pandemic on their care management. This study aimed at filling this gap by describing the prevalence of symptoms suggestive of COVID-19 infection in a large neurological sample of 2,167 individuals. Furthermore, since recent studies stressed the negative impact of COVID-19 outbreak on quality of life outcomes (6), subjective worsening of neurological conditions and effects of social restrictions were also investigated by a dedicated telephone survey.

## Materials and Methods

### Patient Population

Individuals with chronic neurological diseases who were regularly followed at the outpatient clinic of the Department of Neurology at Agostino Gemelli University Hospital Foundation IRCCS-Catholic University of the Sacred Heart in Rome were consecutively enrolled in the study if they had a scheduled visit during the lockdown.

### Inclusion Criteria

Patients with chronic neurological diseases with scheduled visits during the lockdown period, postponed due to social restrictions.

### Exclusion Criteria

Patients were excluded if they or their legal support administrator were unable to provide informed and valid consent at the time of the assessment. Patients with cognitive deterioration and not fluent in Italian were also excluded if a caregiver was not available for the interview.

### Survey Design and Testing

The study was conducted through a telephone survey. Surveys started on April 1, 2020, and ended on April 15, 2020. A dedicated questionnaire, based on current evidence of SARS-CoV-2 (7), was adopted to collect information on symptoms suggestive of COVID-19 in patients with chronic neurological conditions and to evaluate the impact of social restrictions on the perception of illness. Specifically, the survey assessed: (1) Demographic and clinical characteristics, including age at onset, duration of illness, and disability measures (ADL/IADL) (8); (2) COVID-19 related questions, including history of recent travel in endemic areas, direct contacts with COVID-19 confirmed cases (COVID-19+), symptoms suggestive of COVID-19 infection started or worsened in the last 3 months (fever, cough/sore throat, asthenia, dyspnea, myalgia, and hyposmia/hypogeusia), and confirmatory testing for COVID-19 (nasal/pharyngeal swab test results); (3) information related to the impact of COVID-19 on disease burden, including subjective worsening of neurological symptoms, compliance with restrictions and specific effects of restriction measures on the perception of illness (need of urgent neurological care, discontinuation of pharmacological treatment or physiotherapy, difficulties in finding drugs). We chose the last 3 months as the period in which symptoms could be attributed to SARS-CoV-2 infection because the first case of COVID-19 in Italy was confirmed on January 30, 2020.

Comorbid medical conditions, smoking habits and current pharmacological treatments were also investigated. Drug classes potentially interfering with SARS-CoV-2 (9) (i.e., ACE-inhibitors, sartans, NSAIDs, steroids, immunosuppressant drugs) were distinguished from other pharmacological treatments, including specific treatments for neurological disease (i.e., levodopa, anticholinesterase drugs, memantine, xenazine, riluzole, botulinum toxin injections, and antiepileptic drugs).

### Statistical Analysis

All the statistical analyses were carried out using the “Statistical Package for Social Science (SPSS)” program, version 25.0 (IBM Co., Armonk, NY). Collected data were analyzed for normality of distribution using the Kolmogorov-Smirnov test of normality and expressed as mean ± SD (continuous variables) and as frequencies (n, %) for categorical variables according to neurological diagnosis. Univariate correlations were calculated using the non-parametric Spearman correlation coefficient. After adjustment for multiple measures (Bonferroni correction), a *p* < 0.01 was considered statistically significant. The Mann–Whitney and χ^2^ tests were used to assess the significance of the differences between subgroups, as appropriate. For major findings the effect size was also reported.

### Standard Protocol Approvals, Registration, and Patient Consent

The Survey was reviewed and approved by the Agostino Gemelli University Hospital Foundation IRCCS-Catholic University of the Sacred Heart Ethics Committee, Rome. Because of the biological risks related to the pandemic, participants could not timely provide written informed consent. Therefore, during the phone call, verbal consent was obtained for study participation and use of anonymized data (immediate consent), according to information filed with the Ethics Committee. Participants were informed that written consent would be obtained at the first visit in the hospital (deferred consent).

### Data Availability

Upon approved requests, anonymized data will be shared with qualified external researchers.

## Results

Two thousand two hundred and eighty-nine patients were surveyed; 122 participants were excluded for incomplete data, unavailability of legal support administrator at the time of the assessment. Twenty-nine patients (1.3%) refused to participate in the study. The final study sample included 2,167 patients. In the final sample, the male/female ratio was 1,066/1,101 (0.968), mean age was 59 ± 18 and mean illness duration was 12 ± 12 years.

Demographic and clinical characteristics of the sample (Table 1), COVID-19 related questions (Table 2), and information related to the impact of COVID-19 on disease burden (Table 3) are reported for each neurological diagnostic group.

**Table 1:** Demographic and disease characteristics.

**Neurological diagnosis**	**N°**	**Mean age (y)**	**Male subjects**	**Disease duration (y)**	**Age at onset (y)**	**ADL ≤ 3**	**IADL ≤ 4**	**Ambulatory patients**
ALS	84 (4%)	65 ± 11	54 (64%)	4 ± 3	62 ± 11	51 (61%)	57 (68%)	42 (50%)
CI	173 (8%)	76 ± 8	66 (38%)	4 ± 3	72 ± 9	52 (30%)	129 (75%)	146 (84%)
Dystonia	104 (5%)	68 ± 12	26 (25%)	16 ± 10	52 ± 15	1 (1%)	2 (2%)	104 (100%)
Epilepsy	107 (5%)	46 ± 18	47 (44%)	14 ± 13	32 ± 22	6 (6%)	18 (17%)	102 (95%)
HD & TS	100 (5%)	58 ± 13	46 (46%)	9 ± 7	50 ± 15	28 (28%)	57 (57%)	77 (77%)
Headache	97 (4%)	44 ± 16	24 (25%)	21 ± 15	24 ± 14	0 (0%)	2 (2%)	97 (100%)
MS	201 (9%)	45 ± 14	54 (27%)	12 ± 9	33 ± 12	19 (9%)	21 (10%)	172 (86%)
Myasthenia	111 (5%)	60 ± 17	51 (46%)	14 ± 10	47 ± 21	4 (4%)	9 (8%)	108 (97%)
Myopathies	371 (17%)	51 ± 15	198 (53%)	21 ± 12	30 ± 15	31 (8%)	11 (18%)	332 (89%)
Neuropathies	57 (3%)	62 ± 17	45 (79%)	8 ± 10	54 ± 20	6 (11%)	57 (19%)	49 (86%)
PD	255 (12%)	70 ± 11	163 (64%)	10 ± 7	60 ± 13	50 (20%)	113 (44%)	199 (78%)
SD	200 (9%)	56 ± 17	109 (55%)	9 ± 10	47 ± 19	8 (4%)	9 (5%)	192 (96%)
HSP & SCA	68 (3%)	50 ± 15	39 (57%)	21 ± 12	29 ± 18	15 (22%)	31 (46%)	48 (71%)
Stroke	239 (11%)	69 ±14	144 (60%)	0.9 ± 0.4	68 ± 14	61 (26%)	77 (32%)	199 (83%)
Total	2 167	59 ± 18	1,066 (49%)	12 ± 12	47 ± 22	332 (15%)	602 (28%)	1,867 (86%)

*PD, Parkinson disease; HD & TS, Huntington disease and Tourette syndrome; CI, Cognitive Impairment; MS, Multiple Sclerosis; ALS, Amyotrophic Lateral Sclerosis; SD, Sleep Disorder; HSP & SCA, Hereditary spastic paraplegia and Spinocerebellar ataxia*.

**Table 2:** COVID-related variables.

**Neurological diagnosis**	**Flu-related symptoms**						**Nasopharyngeal swab**	
	**Fever**	**Cough/Sore thorat**	**Asthenia**	**Myalgia**	**Dyspnoea**	**Hyspomia/ hypogeusia**	**Performed**	**Positive**
ALS	3 (4%)	6 (7%)	4 (5%)	(2 (2%)	2 (2%)	1 (1%)	2 (2%)	0
CI	11 (6%)	25 (14%)	(14 (8%)	12 (7%)	4 (2%)	11 (6%)	1 (1%)	0
Dystonia	8 (8%)	18 (17%)	7 (7%)	3 (3%)	1 (1%)	0 (0%)	1 (1%)	0
Epilepsy	13 (12%)	16 (15%)	13 (12%)	11 (10%)	6 (6%)	3 (3%)	5 (5%)	0
HD & TS	10 (10%)	23 (23%)	12 (12%)	6 (6%)	5 (5%)	2 (2%)	2 (2%)	0
Headache	17 (19%)	30 (31%)	15 (15%)	12 (12%)	6 (6%)	4 (4%)	3 (3%)	0
Myasthenia	3 (3%)	12 (11%)	10 (9%)	2 (2%)	4 (4%)	1 (1%)	1 (1%)	0
Myopathies	40 (11%)	92 (25%)	30 (8%)	49 (13%)	13 (4%)	4 (1%)	3 (1%)	0
MS	30 (15%)	72 (36%)	39 (19%)	28 (14%)	7 (3%)	6 (3%)	3 (1%)	2 (1%)
Neuropathies	10 (18%)	14 (25%)	10 (18%)	7 (12%)	2 (4%)	2 (4%)	0 (0%)	0
PD	18 (7%)	30 (12%)	16 (6%)	10 (4%)	9 (4%)	31 (12%)	9 (4%)	0
SD	19 (10%)	42 (21%)	20 (10%)	13 (10%)	8 (4%)	8 (4%)	7 (4%)	0
HSP & SCA	2 (3%)	5 (7%)	0 (0%)	0 (0%)	0 (0%)	0 (0%)	0 (0%)	0
Stroke	22 (9%)	38 (16%)	33 (14%)	14 (6%)	11 (5%)	10 (4%)	4 (2%)	0
Total	206 (10%)	423 (20%)	223 (10%)	169 (8%)	78 (4%)	83 (4%)	41 (1.9%)	2 (0.09%)

*PD, Parkinson disease; HD & TS, Huntington disease and Tourette syndrome; CI, Cognitive Impairment; MS, Multiple Sclerosis; ALS, Amyotrophic Lateral Sclerosis; SD, Sleep Disorder; HSP & SCA, Hereditary spastic paraplegia and Spinocerebellar ataxia*.

**Table 3:** Consequences of pandemic COVID-19 on neurology outpatients.

**Neurological diagnosis**	**Subjective worsening of neurological condition**	**Suspension hospital treatments or physiotherapy**	**Difficulty finding drugs**	**Need for urgent consultation**	**Compliance social restriction**	
					**Before March 11th**	**After March 11th**
ALS	40 (48%)	63 (75%)	2 (2%)	12 (14%)	61 (73%)	79 (94%)
CI	82 (47%)	37 (21%)	5 (3%)	33 (19%)	108 (62%)	127 (73%)
Dystonia	31 (30%)	101 (97%)	3 (3%)	1 (1%)	31 (30%)	102 (98%)
Epilepsy	7 (7%)	3 (3%)	5 (5%)	6 (6%)	55 (51%)	88 (82%)
HD & TS	10 (10%)	18 (18%)	8 (8%)	8 (8%)	57 (57%)	97 (97%)
Headache	20 (21%)	8 (8%)	1 (1%)	6 (6%)	48 (49%)	87 (90%)
MS	32 (16%)	53 (26%)	6 (3%)	11 (5%)	88 (44%)	194 (97%)
Myasthenia	12 (11%)	3 (3%)	4 (4%)	14 (13%)	51 (46%)	91 (82%)
Myopathies	33 (9%)	151 (41%)	2 (1%)	16 (4%)	231 (62%)	354 (95%)
Neuropathies	15 (26%)	15 (26%)	1 (2%)	3 (5%)	15 (26%)	50 (88%)
PD	64 (25%)	104 (41%)	21 (8%)	27 (11%)	102 (40%)	236 (93%)
SD	33 (17%)	5 (3%)	8 (4%)	12 (6%)	128 (64%)	108 (54%)
HSP & SCA	5 (7%)	29 (43%)	0 (0%)	1 (1%)	53 (78%)	67 (99%)
Stroke	24 (10%)	51 (21%)	10 (4%)	8 (3%)	134 (56%)	219 (92%)
Total	408 (19%)	641 (30%)	76 (4%)	158 (7%)	1,162 (54%)	1,899 (88%)

*PD, Parkinson disease; HD & TS, Huntington disease and Tourette syndrome; CI, Cognitive Impairment; MS, Multiple Sclerosis; ALS, Amyotrophic Lateral Sclerosis; SD, Sleep Disorder; HSP & SCA, Hereditary spastic paraplegia and Spinocerebellar ataxia*.

### Demographic and Clinical Characteristics

The main demographic and clinical characteristics of the sample are reported in Table 1 for each neurological group. Furthermore, 1,207 individuals reported one concomitant medical condition and 653 participants reported two or more concomitant medical conditions; 844 individuals (39%) reported hypertension, with the highest prevalence among patients affected by stroke (82%), 411 were affected by heart disease (19%), and 233 by lung disease (11%); 270 participants presented with diabetes (13%), 188 reported cancer (9%), 64 chronic kidney diseases (3%), and 220 obesity (10%). Furthermore, 530 patients were current or former smokers (25%).

Among the 1,189 patients who were on drug treatment, 365 (17%) were on ACE-inhibitors, 301 (14%) on sartanics, 164 (8%) on non-steroidal anti-inflammatory drugs (NSAIDs), 154 (7%) on steroids, and 205 (9%) on immunosuppressant/immunomodulatory drugs. Hospital delivered, and infusion therapies, including botulinum toxin and other neurological disease–specific treatments, were reported by 1,387 individuals (64%).

### COVID-19 Related Questions

Distribution of symptoms suggestive of COVID-19 infection and results of confirmatory testing are reported in Table 2 for each neurological group. Sixty-three patients (3%), 10 of whom were cohabitants (0.5%), reported a contact with COVID-19+ individuals, and 58 patients (2.7%) had recently traveled in endemic areas. Forty-one individuals (1.9%) were tested with nasal/pharyngeal swabs, two were COVID-19+ (4.9% of screened patients, 0.09% of the total sample).

The first COVID-19+ was a 57-years-old woman with a 14-year history of secondary progressive multiple sclerosis (ADL score = 2/6) in treatment with azathioprine. During the stay in a rehabilitation clinic, she presented with high fever and fatigue for 2-days, followed by hypogeusia. Symptoms promptly resolved and she did not require hospitalization; nevertheless, she reported a transient worsening of neurological symptoms.

The second COVID-19+ was a 60-years-old woman, smoker, with an 8-year history of relapsing/remitting multiple sclerosis (ADL score = 6/6), on ocrelizumab treatment (last infusion in October 2019). After her mother developed COVID+ pneumonia, she reported moderate fever and cough lasting for 5 days. Thereafter, she spontaneously recovered and did not report any worsening of previous neurological symptomatology.

Correlations between the clinical condition suggestive of COVID-19 infection (at least 3 among fever, cough, asthenia, dyspnea, myalgia, hyposmia in the last 3 months) (10) and subjective worsening of neurological symptoms, disability and compliance to restriction measures are reported in Table 4A. We decided to evaluate these aspects in the patients with clinical conditions characterized by at least three of COVID-symptoms to increase the probability that these subjects were affected by COVID-19. We found a significant positive correlation between COVID-19 symptoms and subjective worsening of neurological symptoms and a negative association between COVID-19 symptoms and ability to walk. Among patients who experienced COVID-19 symptoms and asymptomatic individuals, 28 and 18% reported subjective worsening of neurological symptoms, respectively (*p* = 0.002, *h* = 0.16 – small); 7% of ambulatory subjects and 12% of non-ambulating patients presented with COVID-19 flu symptoms (*p* = 0.001, *h* = 0.18 – small). Finally, in our population, there was no correlation between the suggestive symptoms of COVID-19, either when considered individually or in combination of at least three symptoms, and the usage of steroid or immunosuppressant therapies.

**Table 4:** Reduced Correlation Matrix of univariate analysis [Spearman rho correlation coefficient (significance)].

	**Subjective perception of worsening**	**Total ADL**	**Total IADL**	**Ambulatory patients**	**Compliance to social restriction before March 11th**	**Compliance to social restriction after March 11th**
**A**						
Presence of 3+ flu symptoms	0.067 (0.002)	−0.051 (0.017)	−0.015 (0.484)	−0.068 (0.001)	0.021 (0.334)	0.029 (0.173)
	**Need for urgent neurological visit**	**Total ADL**	**Total IADL**	**Suspension of treatments**	**Difficulty finding drugs**	
**B**						
Subjective Worsening Perception	0.451 (<0.001)	−0.058 (0.007)	−0.075 (0.001)	0.065 (0.002)	−0.031 (0.151)	

### Lockdown Consequences on Disease Burden

Information related to the impact of COVID-19 on disease burden is reported in Table 3 for each neurological group.

Social restrictions were respected by 88% of the participants, ranging from 54% among patients with sleep disorders, to over 95% among patients with movement disorders, multiple sclerosis, and myopathies. One hundred and fifty-eight individuals (7%) needed urgent neurological care, which was deferred due to the lockdown; 641 patients (30%) suspended the hospital treatments (including botulinum toxin injection and infusion treatments of immunomodulatory drugs), physiotherapy or other support interventions, 76 patients (4%) complained about drug unavailability, 408 individuals (19%) reported a subjective worsening of neurological symptoms (Table 3). We reviewed our data about lockdown effects on function and disability in the group of patients aged seventy and older (695 subjects; 32%): 154 patients (22%) reported a subjective worsening of neurological symptoms; 62 individuals (9%) needed urgent neurological care; 222 patients (32%) suspended the hospital treatments, physiotherapy or other support interventions; 35 patients (5%) reported drug unavailability. In the same group, low ADL/IADL scores, indicating higher disability, was detected, respectively, in 180 (26%) and 331 (48%) subjects.

Correlations between subjective worsening of neurological symptoms and specific consequences of social restrictions are reported in Table 4B. Specifically, we found a direct correlation between the subjective worsening of neurological symptoms and need of urgent neurological care (*p* < 0.001, *h* = 0.94 - large) and discontinuation of hospital treatment or physiotherapy (*p* = 0.002; *h* = 0.16). Also low ADL/IADL scores, indicating higher disability, significantly correlated with subjective worsening of neurological symptoms during the pandemic (*p* = 0.007 and *p* = 0.001).

## Discussion

This study aimed to report the prevalence of COVID-19 infection/symptoms and to analyze the impact of restriction measures among patients with chronic neurological disorders. It included over 2,000 patients regularly followed at different services of our outpatient clinic.

Although patients with neurological diseases might be particularly at risk for SARS-CoV-2 (4), in our sample only two patients (0.09%) of 41 tested through nasal-pharyngeal swab (4.9%) received a diagnosis of SARS-CoV-2 infection. Furthermore, these two patients, both affected by multiple sclerosis, presented with a mild clinical picture, not requiring specific antiviral treatments or hospitalization. On April 15, 2020, out of 5,897,000 inhabitants in the Lazio region, 75,584 individuals had been screened through nasal-pharyngeal swab, of whom 5,232 (0.09%) were found to be COVID-19+. Accordingly, the prevalence of COVID-19 infection (positive swab) in our sample (0.09%) was consistent with the one observed in the general screened population. Therefore, our results suggest that chronic neurological disorders may not increase the risk for SARS-CoV-2 infection. Interestingly, the 2 COVID-19+ cases in our sample were both affected by multiple sclerosis and reported minor symptoms. Clinical deterioration during SARS-CoV-2 infection is correlated to a release of pro-inflammatory cytokines and, in our patients, chronic immunosuppressant therapy could have *per-se* mitigated the infection course (11). This possible interpretation need eventual confirmation in larger case series. In our sample a substantial proportion of patients reported fever and cough/sore throat (10 and 20% of the total sample, respectively). Despite it is possible that some of these individuals could have COVID-19, no severe clinical features needing hospitalization or deaths were reported. Previous studies reported that hyposmia is a frequent and specific indicator of SARS-CoV2-infection, when associated with flu-like symptoms, myalgia and asthenia (12, 13). A recent reduction of the sense of smell or taste, or a substantial modification compared to lifetime, were reported in 4% of the sample. Hyposmia is a common non-motor symptom in Parkinson's disease (PD), however in our sample 31 individuals with PD (12%) reported a recent further worsening or new onset of hyposmia.

Concerning the secondary aim of our study, we found a significant association between the presence of COVID-19 symptoms and the subjective worsening of neurological symptoms. This could be due to an objective worsening of underlying medical conditions or to concern about further medical complications in patients with chronic illnesses (14). Nevertheless, our results showed that the subjective worsening of neurological symptoms was associated with the consequences of social restrictions. As a matter of fact, among 408 individuals reporting subjective worsening of neurological symptoms, only 158 patients reported an unmet need for urgent neurological care. Proportions are unevenly distributed among different neurological diagnostic groups exceeding 10% in patients with Parkinsonian syndromes, cognitive impairment, myasthenia, and amyotrophic lateral sclerosis (ALS). We can suppose that this distribution reflects the complex clinical management of these diseases, often requiring a multidisciplinary team. Patients with the above-mentioned syndromes are often in treatment with polypharmacotherapy and with supportive therapies such as neuromotor, speech, and occupational therapies. Unfortunately, during the pandemic these treatments have been interrupted due to social restrictions. In particular, regarding the high percentage of patients affected by ALS that referred a progression of symptoms, we can speculate that this is partly attributable to the natural progression of the disease but could also be due to the impossibility of carrying out planned neurological and pneumological assessments, as well as physiotherapy. In our sample, 1,337 patients were under neurological disease–specific treatments, including botulinum toxin and infusion therapies for neuroinflammatory diseases, headache and other disorders; 30% of these patients could not receive the scheduled treatments due to restrained hospital routine.

Conversely, difficulties in obtaining pharmacological treatments were reported only by a small percentage of patients (8%), the majority in the Parkinson group. Overall, telephone contacts were extremely helpful in reassuring most patients and caregivers and allowed to postpone scheduled medical visits. Not clinically significant differences about lockdown effect on function and disability were detected in the group of patients aged 70 and older. These subjects, as expected, presented a lower ADL/IADL scores.

Finally, the lack of correlation between COVID-symptoms and the usage of steroid or immunosuppressant therapies does not suggest to interrupt or modify these treatments in patients with neurological disorders.

Our study has several limits. First, the present study is not a population-based analysis. Since our hospital is a referral center for rare neurological diseases, some of them were over-represented with respect to the Italian general population (15). At the same time, the broad spectrum of neurological diseases involved in this survey, encompassing subgroups characterized by a particular vulnerability to lockdown such as rare neurological diseases, can well-represent the impact of the pandemic on chronic neurological disorders followed in the Community hospitals and non-reference centers. Furthermore, results showed a high degree of variability in age at onset and illness duration, ranging from few months in the group of patients presenting with stroke, to many decades in the genetic diseases group. Another limitation was the lack of standardized questionnaires for cognition, mood or quality of life. However, all patients underwent a detailed neurological anamnesis which included the evaluation of these clinical aspects. In particular, the worsening of the patient's clinical condition was considered only if clearly distinguished from a worsening of mood or is reported by the caregiver in case of cognitive decline (i.e., Alzheimer disease, PD). Comorbid medical conditions and drug treatment were also unevenly distributed in the cohort. As expected, they were not related to the specific neurological conditions, but to individual risk factors and demographic variables (i.e., hypertension was more frequent among patients with stroke and in older individuals). Conversely, women and men were homogenously represented in the total sample (M/F: 49/51), and sex ratio in the specific diagnostic subgroups was in accordance with previous data in the specific neurological populations (Table 1). Second, even though results suggest that chronic neurological disorders may not increase the risk of COVID, only 41 patients underwent swab (1.9% of the sample). Therefore, the exact number of COVID+ cases among individuals presenting with flu-like symptoms suggestive of SARS-CoV-2 infection could not be precisely established. Moreover, asymptomatic cases may also underestimate the real prevalence of the infection. Third, the survey design required telephone contact rather than face-to-face assessment, as a consequence, the interview may be influenced by uncontrolled and recall bias. Finally, our observations and conclusions are limited by the study's retrospective and cross-sectional design.

In conclusion, our data suggest that chronic neurologic diseases did not increase the prevalence of COVID-19 infection. Lockdown restriction measures were associated with subjective worsening of neurological symptoms and may have exacerbated the burden of neurological disorders.

## Data Availability Statement

The raw data supporting the conclusions of this article will be made available by the authors, without undue reservation.

## Ethics Statement

The studies involving human participants were reviewed and approved by Universitá Cattolica del Sacro Cuore Ethics Committee, Rome. Written informed consent for participation was not required for this study in accordance with the national legislation and the institutional requirements.

## Author Contributions

CP and ED designed and conceptualized study and collected and analyzed the data. GPri interpreted the data, revised the manuscript for intellectual content, and drafted the manuscript. PC and AB designed and conceptualized study, analyzed the data, and revised the manuscript for intellectual content. DJ, MLui, GF, CV, MLuc, VB, MMo, VG, GD, AE, CM, MMi, DQ, ERi, SS, GSi, SBe, SBo, FB, RD, AD, DG, TI, MRL, JM, AKP, AP, MP, GPre, VR, ERo, AR, MR, CS, IS, GSp, MS, LT, and PZ collected and interpreted the data and revised the manuscript for intellectual content.

## Conflict of Interest

The authors declare that the research was conducted in the absence of any commercial or financial relationships that could be construed as a potential conflict of interest.
